# Removal of Microplastics from Drinking Water by *Moringa oleifera* Seed: Comparative Performance with Alum in Direct and in-Line Filtration Systems

**DOI:** 10.1021/acsomega.5c11569

**Published:** 2026-01-19

**Authors:** Gabrielle S. Batista, Victoria A. S. Ferreira, Luiz G. R. Godoy, Rodrigo B. Moruzzi, Soroosh Sharifi, Adriano G. dos Reis

**Affiliations:** † São Paulo State University (UNESP), Institute of Science and Technology, Environmental Engineering Department, São José dos Campos 12247-016, Brazil; ‡ University of Birmingham, School of Engineering, Department of Civil Engineering, Edgbaston, Birmingham B15 2TT, U.K.

## Abstract

In this study, the removal efficiency of aged polyvinyl chloride microplastics (Aged-PVC MPs) from low-turbidity drinking water using *Moringa oleifera* seed saline extract (MOS-SE) and aluminum sulfate (alum) in direct filtration (coagulation–flocculation–filtration) and in-line filtration (coagulation–filtration) systems is investigated. Aged-PVC MPs (15 mg/L, D50 = 15.0 μm) and humic acid (10 mg/L) were spiked into synthetic water to evaluate removal performance across pH 5.0–8.0. The optimal conditions achieved >98% turbidity removal with 30 mg/L MOS-SE and 9 mg/L alum at a pH of 6.0, corresponding to 98.5% and 98.7% of Aged-PVC MP removal, respectively, as confirmed by scanning electron microscopy particle counting. Although nonintrusive floc imaging revealed differences between coagulated (43–46 μm) and flocculated aggregates (61–66 μm), in-line filtration performs equivalently to direct filtration in terms of MP removal, demonstrating that direct filtration’s flocculation step was unnecessary. MOS-SE exhibited superior performance across broader pH ranges (5.0–8.0) compared with alum (5.0–7.0). While MOS-SE increased dissolved organic carbon because of residual organic matter, it effectively reduced specific ultraviolet absorbance (SUVA) by 88%, indicating efficient removal of aromatic natural organic matter. These findings demonstrate the viability of *Moringa oleifera* as a sustainable alternative for MP removal in drinking water treatment via in-line filtration.

## Graphical abstract

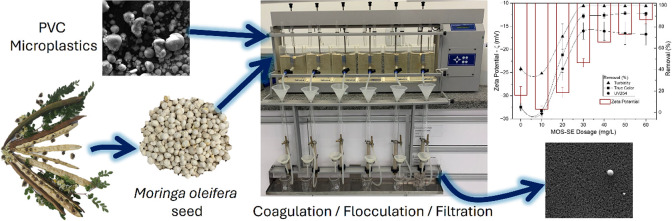

## 1 Introduction

Plastic pollution is now widely recognized as a critical global environmental challenge, with profound threats to aquatic and terrestrial ecosystems.^1^ Among emerging contaminants, microplastics (MPs; 0.1 μm–5 mm) are of growing concern because of their ubiquity, persistence, and potential adverse effects on human and ecosystem health.^2,3^ MPs are routinely detected in drinking water sources, food, and atmospheric fallout, raising public health questions amid evidence of significant daily ingestion rates by humans.^4–6^

Conventional drinking water treatment plants (DWTPs) employing coagulation–flocculation–sedimentation (CFS) can remove a significant fraction of MPs but show broad efficiency variability (typically 40–70%), with incomplete removal often reported.^7^ Process optimization remains an open challenge, particularly regarding the interaction of MPs with coagulants under diverse water quality scenarios.^7–9^ Increasing regulatory scrutiny and health concerns over the use of aluminum and iron-based coagulants—linked to nonbiodegradability, residual toxicity, and disease risk—have intensified the search for sustainable alternatives.^10,11^

Recent advances have highlighted natural coagulants (NCs) from plant, animal, and microbial origins as promising eco-friendly substitutes for synthetic agents.^12^ Of these, *Moringa oleifera* seed extracts are the most extensively studied, with some few reports confirming MP removal efficiencies exceeding 90% under optimized process conditions.^11,13,14^ However, critical research gaps persist, notably in the assessment of NCs within alternative process configurations such as direct filtration (coagulation–flocculation–filtration) and in-line filtration (coagulation–filtration). Although pilot studies have validated the feasibility of NCs in hybridized or CFS frameworks, their performance under low-turbidity, rapid granular filtration regimes and their concomitant effects on natural organic matter (NOM) and specific ultraviolet absorbance (SUVA) have been infrequently reported.

Conceptually, granular filtration is a two-step process comprising particle transport to the collector (filter grain) surface, followed by particle attachment (adhesion). Although transport is governed by physical mechanisms such as interception and sedimentation, successful attachment depends on a complex balance of surface interaction forces. For a particle to be retained, the net adhesive forces—primarily the attractive London–van der Waals force (FvdW) and the typically repulsive electric double layer force (FDCE)—must overcome the hydrodynamic drag and lift forces exerted by the fluid flow. The primary role of coagulation is therefore to reduce electrostatic repulsion (FDCE), which constitutes the main energy barrier to particle adhesion in water filtration.^15,16^

This study addresses these research gaps by systematically evaluating the performance of *M. oleifera* seed extract and alum in both direct and in-line filtration for MP removal from low-turbidity drinking water. Distinctively, the work integrates comparative nonintrusive floc growth imaging and tracks organic matter and SUVA removal efficiency. Through this focus, the aim of this study is to elucidate the applicability and mechanisms of plant-based coagulants in contemporary treatment configurations, contributing critical data to the emerging field of sustainable MP remediation in potable water systems.

## 2 Materials and Methods

### 2.1 Polyvinyl Chloride (PVC) Microplastics

PVC microplastics (MPs) were selected because they are among the most hazardous MP types owing to their mutagenic and carcinogenic potential^17^ and their documented prevalence in both surface freshwater and treated water following drinking water treatment processes.^18,19^ In addition to that, PVC microplastics represent 12.8% of total world plastic production in 2024, ranking third only behind polyethylene and polypropylene in production volume.^20^ Virgin PVC MPs (sieved to 150 mesh) were obtained from Niox Comercial Importadora Ltd. (Brazil).

Artificial aging of the MPs was performed via UV irradiation, a procedure that expedites weathering relative to natural processes and replicates environmentally relevant aged MP properties, as established for PVC MPs.^21–23^ Virgin PVC MPs were distributed in glass Petri dishes, aged in a custom-made UV chamber maintained at 35 °C, and exposed to six UV–C lamps (λ = 254 nm, 15 W) for 720 h, after which the samples were homogenized every 24 h. Aged-PVC MPs were stored in a dry, dark environment for subsequent experiments. The particle size distribution was characterized using a CILAS 1190 Particle Size Analyzer, revealing an overall range of 0.04–90 μm, with D50 = 15.0 μm and D90 = 40.3 μm (see Figure S1 in the Supporting Information). The removal efficiency of rapid granular filtration is highly dependent on MP size, with smaller particles (<45 μm) posing major challenges for removal, as consistently reported in the literature.^16,24^

### 2.2 Synthetic Water Preparation

The model test water was prepared by spiking 15 mg/L Aged-PVC MP and 10 mg/L humic acid (HA) (Sigma–Aldrich, technical grade) into tap water. HA was selected to simulate high-molecular-weight, hydrophobic natural organic matter (NOM) found in most surface waters (50–60% of surface water dissolved organic matter) with reproducible physicochemical properties that challenge coagulation and filtration processes.^25,26^ Both raw (well-derived tap) and synthetic water samples were characterized for turbidity (Policontrol AP2000 nephelometer), pH and alkalinity (Metrohm 913 pH meter), electrical conductivity (Tecnofon mCA150 conductivity meter), apparent and true color (Policontrol Aquacolor Cor; Fanem Baby I 206-BL centrifuge), UV_254_ (ThermoScientific Genesys 50 spectrophotometer), and dissolved organic carbon (DOC) concentration (Shimadzu TOC-VCPN Total Organic Carbon Analyzer), following the Standard Methods for the Examination of Water and Wastewater.^27^ The results are listed in Table 1. The increased turbidity, DOC, apparent/true color, and UV_254_ in the synthetic water are attributed to the addition of Aged-PVC MP and HA. A target turbidity near 15 NTU was established—which is appropriate for direct and in-line filtration schemes.^15^ The remaining parameters did not differ significantly (95% confidence) between the two water matrices.

**Table 1:** Characterization of Raw Water (Tap Water) and Synthetic Water (15 mg/L Aged-PVC MPs and 10 mg/L HA in Tap Water)

Parameter	Tap water	Synthetic water
Turbidity (NTU)	<0.1	14.9 ± 2.3
pH	7.1 ± 0.2	7.2 ± 0.2
Alkalinity (mg CaCO_3_/L)	23.4 ± 1.5	23.3 ± 1.5
Electrical conductivity (μS/cm)	54.6 ± 3.8	55.5 ± 3.8
Apparent color (HU)	1.0 ± 0.7	70.5 ± 11.9
True color (HU)	<0.1	58.1 ± 9.3
UV_254_ (cm^–1^)	0.004 ± 0.008	0.156 ± 0.018
DOC (mg/L)	<0.01	1.84 ± 0.01

### 2.3 Quantification of MPs

Aged-PVC MPs were quantified by complementary indirect and direct approaches. Turbidity reduction was used as an indirect, rapid, and cost-effective proxy for MP removal, given the strong, established correlations between MP concentration and turbidity across several studies.^14,28,29^ Calibration curves were constructed by varying the Aged-PVC MP concentration (with HA) and recording the corresponding turbidity values, yielding a high correlation (*R* ^2^ = 0.99781; Figure S2 in the Supporting Information).

In addition, for experimental conditions with maximal turbidity removal, direct quantification was conducted by particle counting via scanning electron microscopy (SEM). Despite the labor intensity and limited sample throughput, SEM enables precise enumeration and sizing. The protocol, based on,^30^ involved filtering 100 mL aliquots using nitrocellulose membrane filters (47 mm diameter, 0.45 μm pore, GVS, USA), followed by oven drying at 30 °C for 2 h and storage in sealed Petri dishes under desiccation until SEM analysis (Inspect S50—FEI). The membranes were gold-coated (Quantum Model Q150R ES). Random field images were acquired, and particles were automatically quantified (size, count) using Image-Pro-Plus software, scaling to the full membrane area and then to a 1 L sample volume.

### 2.4 Jar Test Protocol

Jar tests were performed with a Policontrol FlocControl III apparatus comprising six square jars (12 × 12 cm), each fitted with a direct granular sand filtration unit. The filtration system consisted of six 19 mm diameter columns, each packed with 17 cm of sand (effective size: 0.47 mm; uniformity coefficient: 1.5; Figure S3 in the Supporting Information).

Direct filtration (coagulation–flocculation–filtration) and in-line filtration (coagulation–filtration) strategies were compared. In both cases, coagulation was applied at a velocity gradient of 1000 s^–1^ for 25 s. For direct filtration, flocculation followed (20 s^–1^, 25 min). Rapid sand filtration for both methods was performed at a flow rate of 9.4 ± 2.2 cm/min. The first 10 min of filtrate (ripening phase) was discarded, after which the samples were collected for turbidity, true color, UV_254_, and DOC analyses. Specific ultraviolet absorbance (SUVA) was calculated to infer the NOM composition according to eq 1:^31^

1 $$SUVA=\frac{UV_{254}}{DOC}\times 100$$

All trials were conducted in triplicate at 24.7 ± 1.1 °C.

The primary coagulants used were saline extract of *Moringa oleifera* seed (MOS-SE) and aluminum sulfate (alum). Coagulants were dosed independently for comparative benchmarking. *M. oleifera* seeds were collected in São José dos Campos, Brazil (23°11′39.8″S 45°55′12.9″W). MOS-SE was prepared by dehusking, milling, sieving (max 600 μm), and extracting with 1 M NaCl to obtain a 0.8% w/w solution, which was subsequently filtered through GF52/C fiberglass (1.2 μm, HNM). The protein content was determined by Kjeldahl;^27^ conversion factor 6.25, where 10 mg/L MOS-SE corresponds to 2.6 mg/L seed protein. The extract had a pH of 6.0 and a zeta potential of +29.0 ± 1.0 mV. The dosages of MOS-SE tested were 0 (control), 10, 20, 30, 40, 50, and 60 mg/L.

Aluminum sulfate [Al_2_(SO_4_)_3_·18H_2_O, ≥98% purity, Êxodo Científica, Brazil] was prepared as a 0.2% w/w stock; 10 mg/L corresponds to 0.79 mg/L Al^3+^. The doses of aluminum sulfate tested were 0 (control), 3, 6, 9, 12, and 15 mg/L.

For in-line filtration, the coagulation pH for each dose was set across 5.0, 6.0, 7.0, and 8.0, and 0.1  M NaOH or HCl was used to identify the optimal removal conditions. For direct filtration, a pH that matched the optimal in-line filtration results without excessive adjustment—pH 6.0—was chosen. Zeta potential at pH 6.0 was assessed using a Litesizer DLS 700 (Anton Paar).

Control experiments with 1 M NaCl (10–60 mg/L, pH 6.0) without *M. oleifera* seeds were included to determine the effects of intrinsic saline on MP removal.

### 2.5 Ives Filterability Index

Filtration performance was evaluated using the Ives filterability index (IFI), according to eq 2:^32^

2 $$IFI=\frac{HC_{e}}{C_{a}tv}$$

where *H* is the head loss (cm), *C_e_* is the average effluent quality, *C_a_* is the average influent quality, *t* is the filtration time (min), and *v* is the approach velocity (cm/min). IFI is dimensionless; here, *C_e_* and *C_a_* represented turbidity (NTU), which correlated well with the MP and HA concentrations (Figure S2 in the Supporting Information).

The IFI is a valuable dimensionless parameter designed as a simple and quick preliminary test to assess the effectiveness of different filtration conditions or media types. A lower IFI value indicates improved filterability, reflecting a combination of low head loss, high particle removal, and a high filtration rate. This makes the index a convenient tool for comparing the relative performance of the systems under investigation.^30^

### 2.6 Nonintrusive Floc Imaging

Floc sizes were assessed nonintrusively at the end of coagulation (in-line) and after flocculation (direct filtration). Imaging was performed for the best Aged-PVC MPs removal conditions for alum and MOS-SE. Images were captured in situ with a high-speed camera (Miro EX-4), positioned perpendicular to a laser light sheet for enhanced floc contrast (Figure S4 in the Supporting Information). Floc sizing followed the procedure in,^33^ extracting 100 frames (10 s at 10 Hz, 800 × 600 pixels), processed in Image-Pro-Plus and analyzed in Microsoft Excel. Manual binarization improved segmentation. Only flocs ≥32.35 μm (≥9 pixels) were analyzed, as per recommended cutoffs.^34^ Floc size was auto-measured based on segmented area.

### 2.7 Statistical Analysis

Data were compared across treatments using one-way ANOVA and Tukey’s multiple comparison test, with significance at *p* < 0.05. The Shapiro–Wilk test was used to check data normality before statistical testing.

## 3 Results

### 3.1 In-Line Filtration: Effects of Coagulant Dosage on the Zeta Potential and Removal Efficiency at pH 6.0

The removal efficiencies for turbidity, true color, and UV_254_, along with the effect on zeta potential, obtained by varying the MOS-SE and alum dosages in in-line filtration at pH 6.0 ± 0.1 are shown in Figure 1. The removal of Aged-PVC MPs was assessed via turbidity reduction, with an initial concentration of 15 mg/L corresponding to 14.9 ± 2.3 NTU. Filtration without added coagulant (0 mg/L) resulted in partial turbidity removal (40.6 ± 2.1%)—thus Aged-PVC MPs—(Figure 1a,b). This partial removal, attributable to physical retention and straining by sand media, is primarily limited to particles larger than void spaces between grains, typically 30–80 μm for rapid sand filters with 0.50 mm effective grain size,^15^ which is consistent with the granulometric analysis for Aged-PVC MPs (Figure S1 in the Supporting Information)—approximately 20% of particles exceed 30 μm. Nondestabilized particles smaller than the intergranular voids pass through, accounting for approximately 60% of the turbidity remaining (and thus Aged-PVC MPs) in the effluent without coagulant. True color (4.9 ± 1.7%) and UV_254_ (5.1 ± 3.6%) removal were low in the absence of coagulant. UV_254_ absorption provides information on aromatic organic compounds present, as simulated by HA addition.^35^

**Figure 1:**
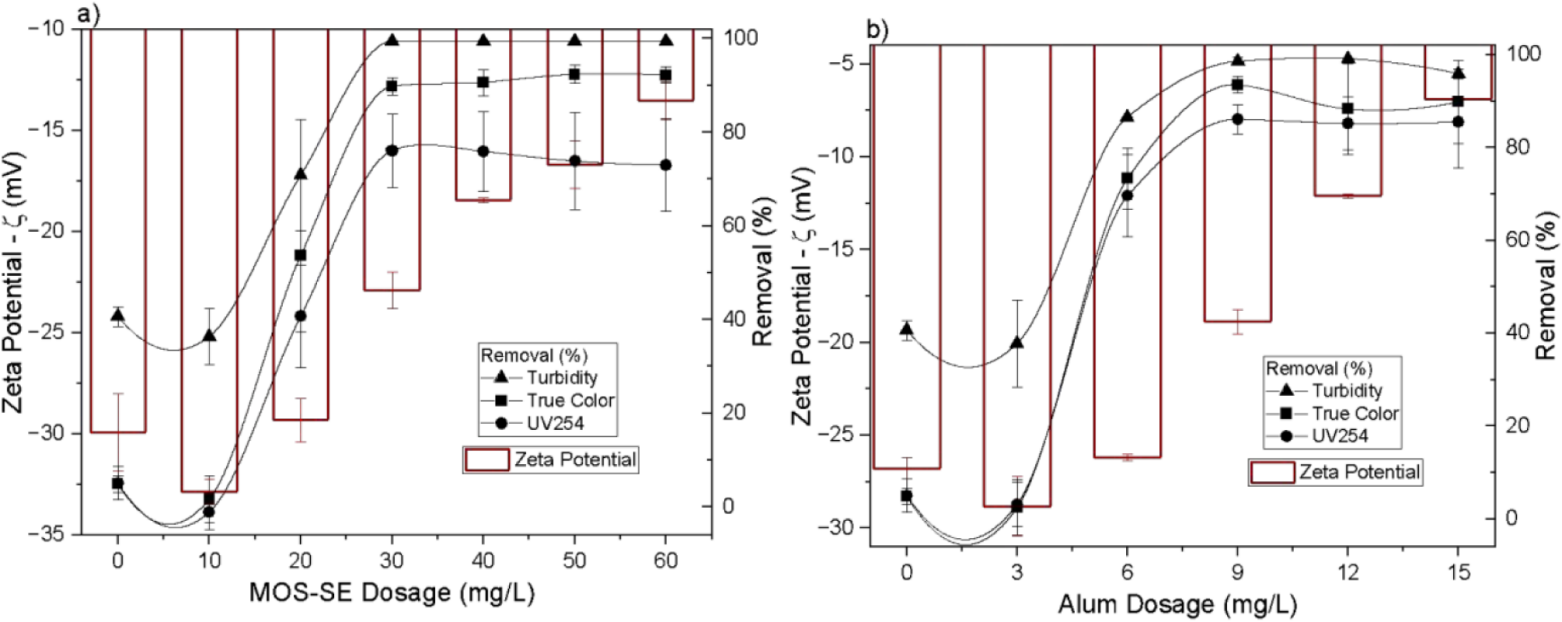
Influence of increasing MOS-SE (a) and alum (b) dosages on the suspension zeta potential and removal of turbidity, true color, and UV_254_ after in-line filtration at pH 6.0. The error bars represent the standard deviations.

Increasing coagulant dosage yielded marked improvement in Aged-PVC MPs removal, reaching 99.4 ± 0.1% turbidity reduction (residual turbidity < 0.1 NTU) at 30 mg/L MOS-SE and 98.6 ± 0.8% (0.2 ± 0.1 NTU) at 9 mg/L alum, with no significant difference at 95% confidence. With MOS-SE, removals for true color and UV_254_ reached 92.4 ± 1.9% and 76.1 ± 7.9%, indicating effective humic acid removal. Alum achieved a statistically comparable true color removal (93.5 ± 1.8%) but significantly higher UV_254_ removal (86.1 ± 3.1%). Improved removal was attributed to destabilization of negatively charged suspended particles and NOM by positive charges from MOS-SE (zeta potential +29.0 ± 1.0 mV). Increased MOS-SE and alum dosages elevated mean zeta potential from −29.9 ± 1.9 mV (synthetic water, 0 mg/L) to −13.5 ± 0.9 mV (60 mg/L MOS-SE) and −6.9 ± 1.4 mV (15 mg/L alum), favoring coagulation via adsorption and charge neutralization. Subsequent aggregation and diminished electrostatic repulsion promoted retention and deposition on negatively charged sand by straining (larger particles) and attachment (smaller particles). This shift toward a more neutral zeta potential indicates compression of the electric double layer (DCE), which critically lowers the electrostatic energy barrier that prevents particles from approaching both each other and the collector surfaces. According to DLVO theory, this reduction in the repulsive force (FDCE) allows the universally present and attractive London–van der Waals forces (FvdW) to become dominant at close separation distances, thereby enabling stable particle attachment to the sand grains. This physicochemical adhesion is thus the principal mechanism for retaining particles smaller than the interstitial pores of the filter.^28,36,37^ A schematic of the Aged-PVC MPs and humic acid removal mechanism by rapid granular filtration with MOS-SE and alum is shown in Figure 2.

**Figure 2:**
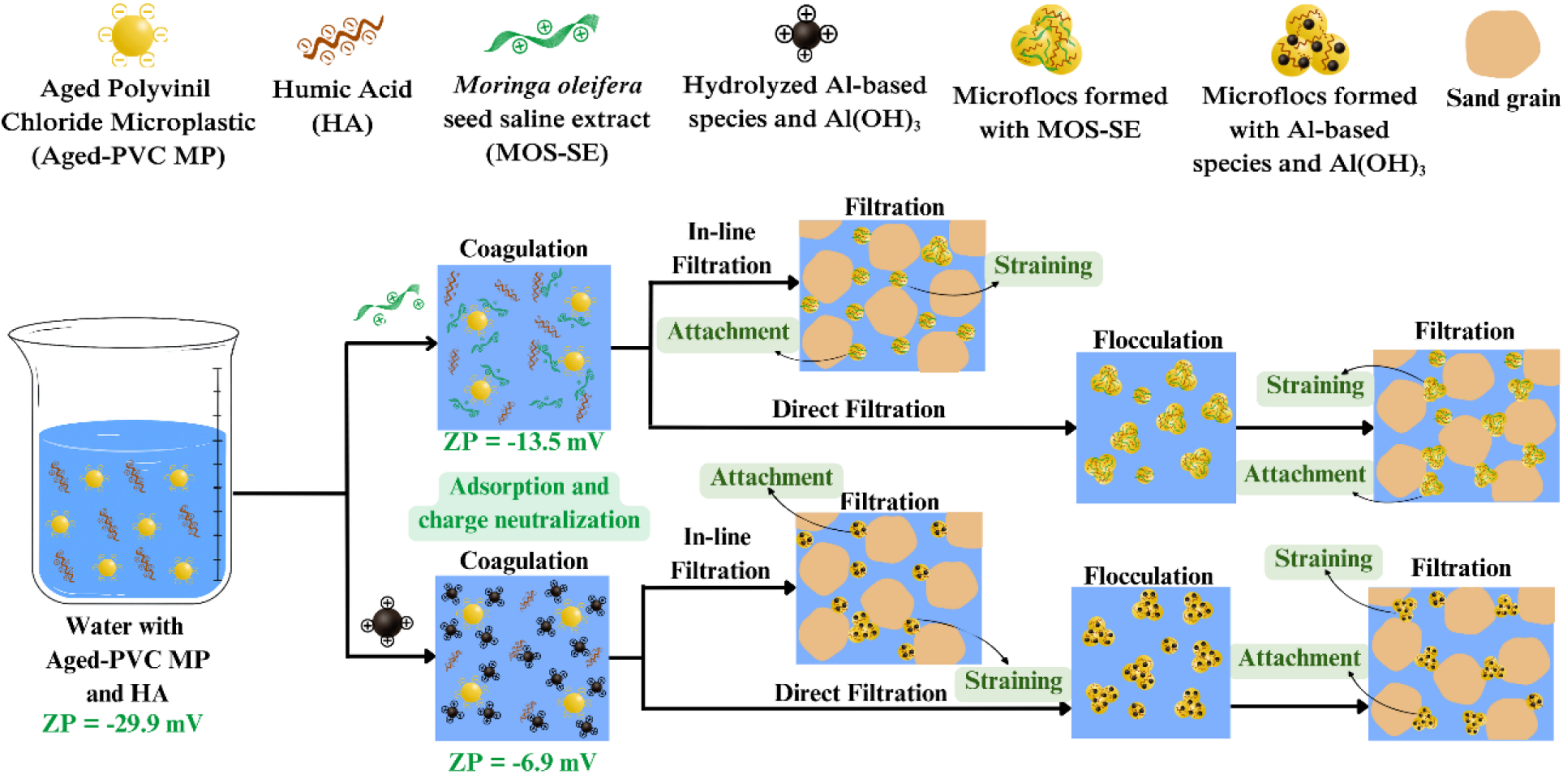
Schematic of Aged-PVC MP and humic acid removal mechanisms using MOS-SE and alum in direct and in-line filtration.

Arenas et al. (2022)^28^ demonstrated that polystyrene (PS) nanoplastics (124 ± 38 nm, 10 mg/L) removal by sand filtration (in-line filtration) increased from 54.3% to 99.2% when polyaluminum chloride (PAC) was added (0.36 mg/L Al^3+^), with zeta potential shifting from −12.0 ± 0.8 mV to −2.9 ± 0.5 mV. The increase in removal efficiency was attributed to the reduction in the surface charge of the PS nanoplastic particles, which significantly improved their retention in the filter media, suggesting that charge neutralization was the dominant coagulation mechanism. Similarly, Na et al. (2021)^37^ found that PS MPs >20 μm were retained by straining, whereas attachment dominated for MPs < 20 μm via rapid granular filtration; the zeta potential increased from −40 mV to +1.9 mV after 10 mg/L aluminum chloride hexahydrate addition at pH 6.0.

Experiments with 1 M NaCl alone across the studied dosages revealed no significant improvement in turbidity, true color, or UV_254_ removal compared with the control group (Figure S5 in the Supporting Information), demonstrating that NaCl solution alone lacks coagulant activity but supports MOS-SE action.^38^

### 3.2 Comparison of Direct vs in-Line Filtration at pH 6.0

The mean aggregate sizes formed after coagulation and flocculation at optimal dosages (30 mg/L MOS-SE, 9 mg/L alum, pH 6.0) are shown in Figure 3. Representative nonintrusive images are shown in Figure 4. The mean aggregate sizes postcoagulation were statistically similar for MOS-SE (43.5 ± 12.0 μm) and alum (46.2 ± 12.8 μm). An additional 25 min of flocculation in direct filtration resulted in statistically significant increases to 61.4 ± 27.3 μm (MOS-SE) and 65.5 ± 32.8 μm (alum). The mean increase in aggregation (41%) after flocculation is considered modest, as anticipated for the charge-neutralization/adsorption mechanism. Godoy et al. (2025)^14^ reported an average floc size of 63.5 μm with MOS-SE-coagulated virgin PVC MPs after 21 min of flocculation, which is consistent with these findings. No reports were found in the literature on floc size growth by alum via charge neutralization. However, Godoy et al. (2024)^39^ evaluated the floc growth of virgin PVC MPs after coagulation with alum (20 mg/L; pH 6.5) via sweep coagulation, and after 21 min of flocculation, the average floc size was 282 μm, which corresponds to an average floc size more than four times greater than that found in this study. Sweep coagulation is applicable to sedimentation rather than rapid filtration. Excessive Al­(OH)_3_ precipitate generation by sweep coagulation compromises filter operation in direct or in-line modes, reaffirming the benefits of charge-neutralization/adsorption for granular filtration.^15^

**Figure 3:**
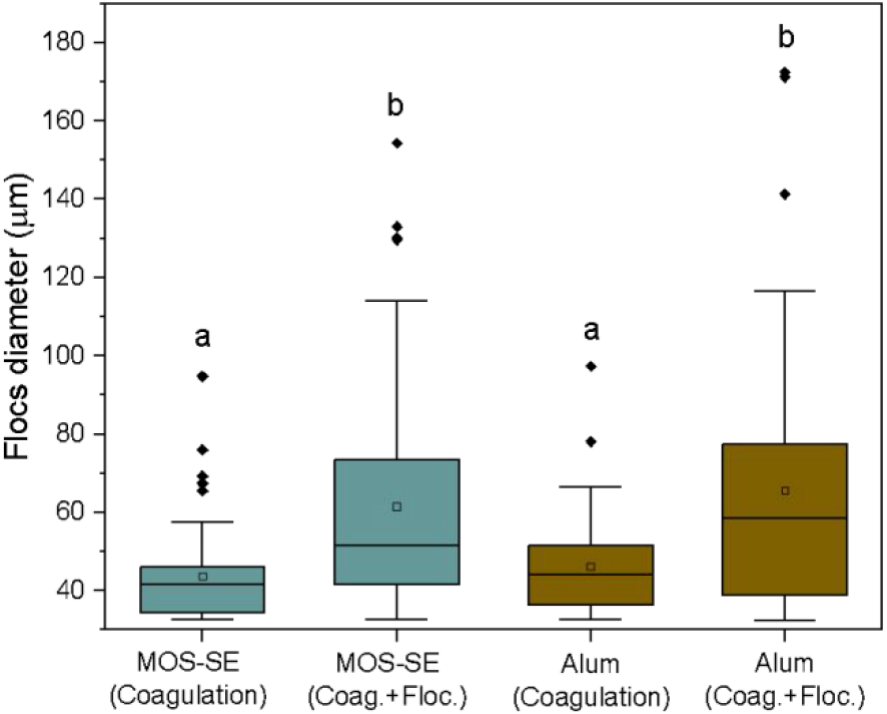
Box plots from nonintrusive image analyses showing floc diameter (μm) of Aged-PVC MPs and HA after coagulation with MOS-SE (30 mg/L, pH 6.0) or alum (9 mg/L, pH 6.0) and after coagulation plus 25 min flocculation. The mean result is denoted by “□”. Boxes with identical letters indicate means without significant difference (*p* ≤ 0.05, Tukey post hoc).

**Figure 4:**
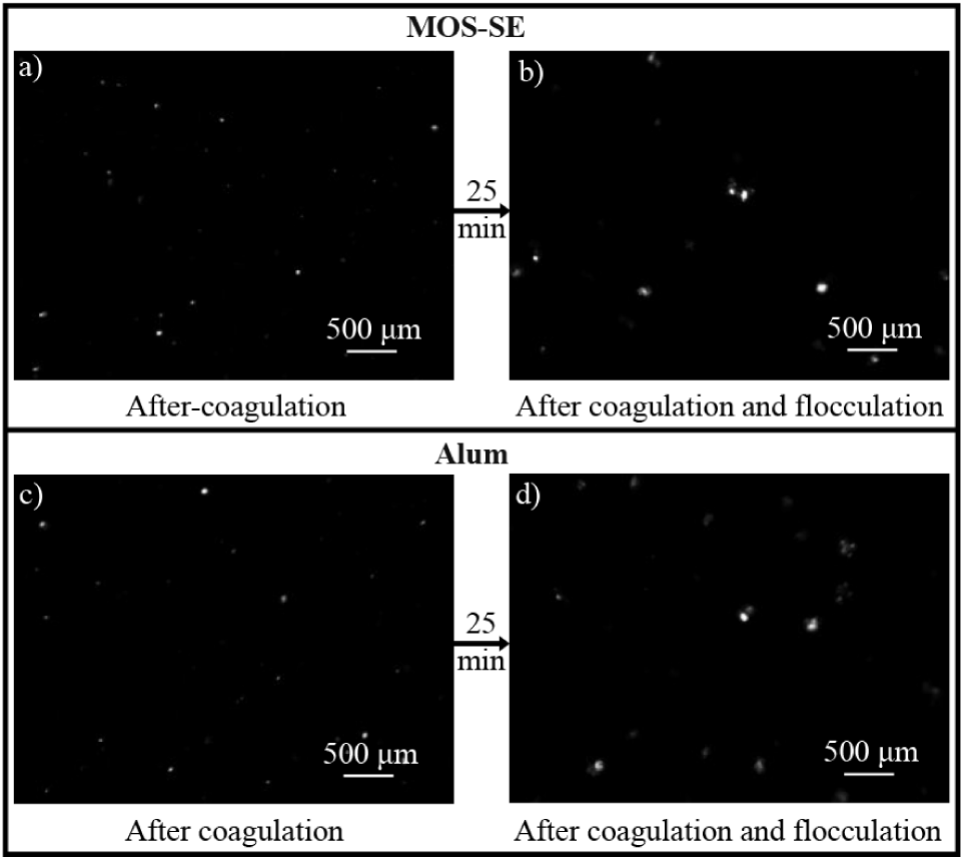
Examples from nonintrusive image analysis (floc growth monitoring): Aged-PVC MPs and HA coagulated with MOS-SE (30 mg/L, pH 6.0) after coagulation (a) and after flocculation (b) and with alum (9 mg/L, pH 6.0) after coagulation (c) and after flocculation (d).

The increase in aggregate size after flocculation likely improves retention by straining versus attachment, as larger aggregates are physically retained within sand voids, whereas smaller particles preferentially adhere to collector surfaces.^37^ The removal mechanism for Aged-PVC MPs and HA by direct filtration with MOS-SE and alum is shown in Figure 2. The necessity of flocculation is typically determined empirically.^15^

A comparative analysis of turbidity, true color, and UV_254_ removal for direct and in-line filtration at a coagulation pH of 6.0 ± 0.1 is presented in Figure 5. Aged-PVC MP removal was tracked by reduced turbidity (Figure S2 in the Supporting Information calibration). Low removal in the absence of coagulant addition (0 mg/L) is attributed solely to physical retention mechanisms during in-line or direct filtration, as previously discussed in Figure 1. No statistically significant differences in removal efficiency were observed between direct and in-line filtration with either MOS-SE or alum, despite increased aggregate sizes postflocculation (Figure 3), indicating that a 25 min flocculation step is unnecessary under these conditions. Thus, as shown in Figure 5, in-line filtration is effective and sufficient for removing Aged-PVC MPs and HA, supporting the results of subsequent studies focused solely on in-line filtration. Although straining becomes more effective for larger aggregates, the results suggest that for the conditions studied, the initial destabilization achieved during coagulation was sufficient to facilitate highly efficient removal via the attachment mechanism. Once the repulsive energy barrier is overcome, the smaller, coagulated particles can adhere effectively to the filter media, making the formation of larger flocs for enhanced straining redundant. It was not found published studies comparing MP removal by direct versus in-line filtration, but Ribeiro et al. (2019)^40^ compared *M. oleifera* seed as a coagulant (30 mg/L, pH 6.7) for low-turbidity (25.4 ± 1.1 NTU) synthetic kaolin water via both modes and reported that additional flocculation did not increase turbidity removal and that in-line filtration minimized DWTP costs.

**Figure 5:**
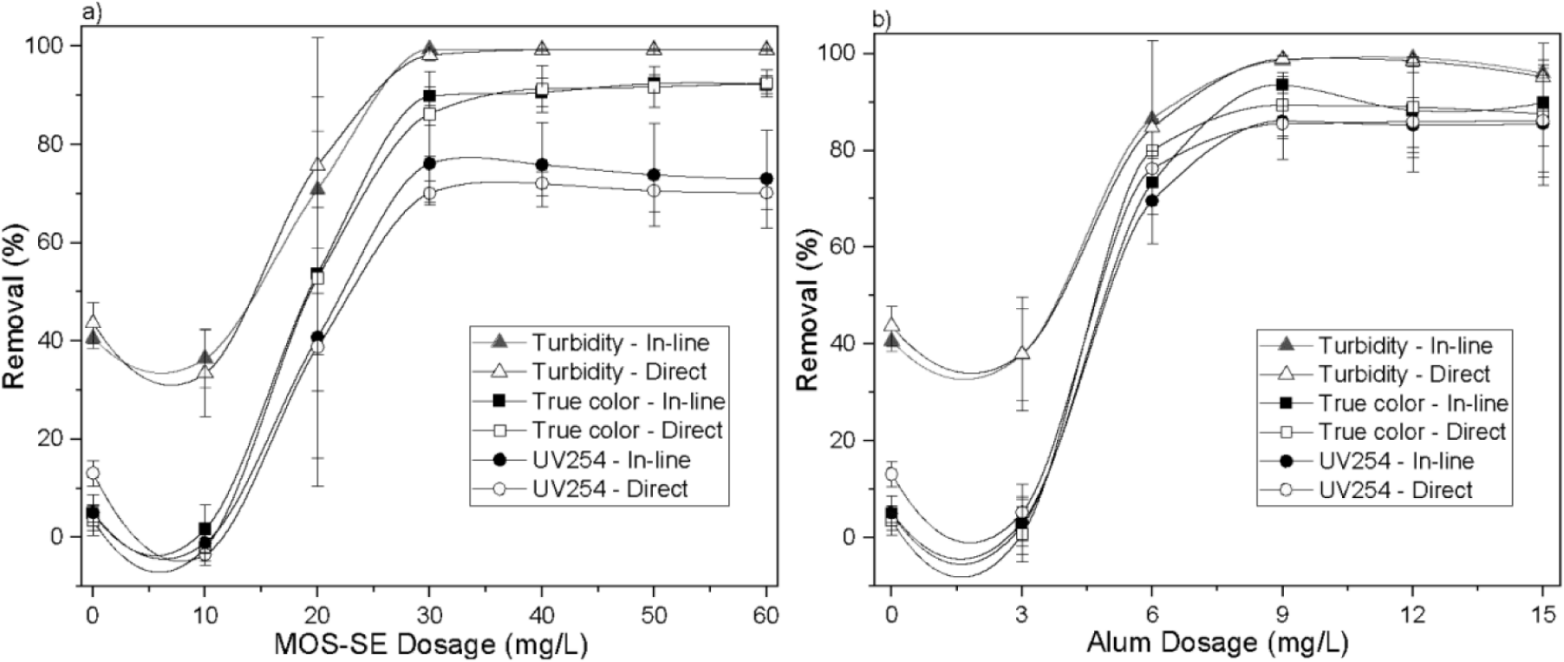
Comparison of turbidity, true color, and UV_254_ removal after in-line and direct filtration at pH 6.0 using various dosages of (a) MOS-SE and (b) alum. The error bars represent standard deviations.

This finding is particularly relevant given that both direct and in-line filtration are typically employed for higher-quality raw water with a turbidity of 15 NTU or less, a condition that is simulated by the synthetic water in this study. The elimination of a dedicated flocculation unit, as in the in-line configuration, therefore represents a significant opportunity for process simplification and cost reduction in plants treated with low-turbidity water.

### 3.3 pH and Coagulant Dosage Effects on in-Line Filtration

The removal efficiencies for turbidity, true color, and UV_254_ using alum and MOS-SE at varying pH values and dosages are shown in Figure 6. The pH values refer to those measured after coagulation. Aged-PVC MP removal was evaluated by reduction in turbidity.

**Figure 6:**
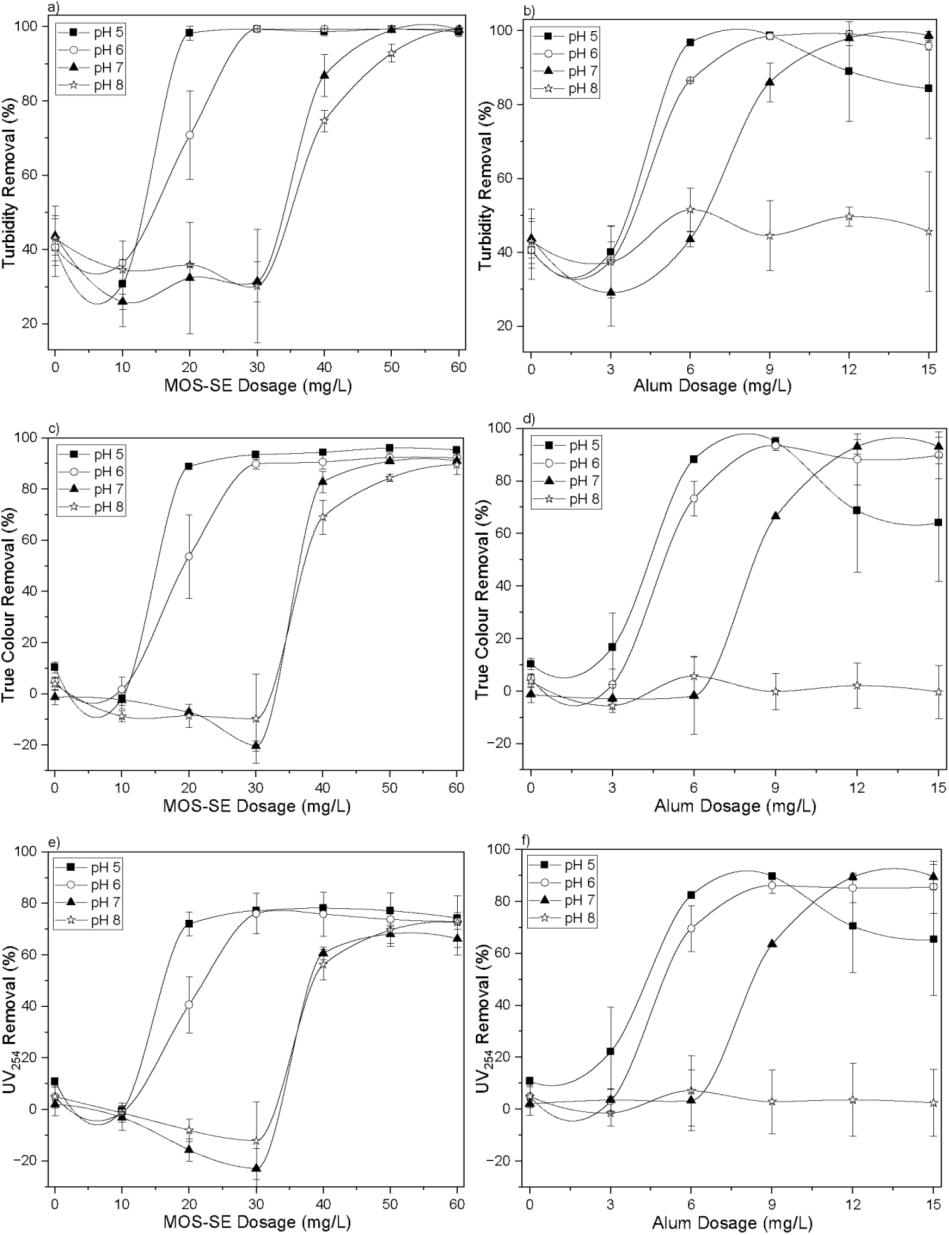
Results after in-line filtration with Aged-PVC MPs and HA using MOS-SE and alum for turbidity removal (a: MOS-SE, b: alum), true color removal (c: MOS-SE, d: alum), and UV_254_ removal (e: MOS-SE, f: alum). The error bars represent standard deviations.

In the absence of MOS-SE or alum, no significant changes in turbidity, true color, or UV_254_ were detected across pH 5.0–8.0, similar to the results at pH 6.0 (Figure 1).

Both MOS-SE and alum were effective at removing suspended particles (via turbidity, Figure 6a,b) and high-molecular-weight NOM (via true color, Figure 6c,d, and UV_254_, Figure 6e,f). The removal patterns were similar across dosages and pH.

With MOS-SE, high Aged-PVC MP removals (>99%) occurred at all pH values (5–9) for dosages ≥20 mg/L (Figure 6a). Increasing pH required higher MOS-SE dosages for effective removal; ≥20 mg/L sufficed at pH 5.0, whereas 60 mg/L was necessary at pH 8.0. Trends for true color (Figure 6c) and UV_254_ (Figure 6e) were comparable. This phenomenon likely reflects the isoelectric point (pI) of *M. oleifera* cationic proteins (pI ≈ 10–11;^41^). The closer the pH to the pI, the fewer positive charges in MOS-SE, necessitating higher dosage for destabilization of negatively charged particles. Lester-Card et al. (2023)^42^ observed consistent removal efficacy with MOS-SE in oily steelworks wastewater (3.0–11.0 pH, 50 mg/L), but reported higher removal and zeta potential at acidic pH (3–5). The improved coagulation-flocculation using the natural coagulant at more acidic pH values was attributed to the cationic nature (positive charge) of the seed proteins under these conditions. Vega Andrade et al. (2021)^36^ evaluated the removal of turbidity from a tertiary sanitary effluent using *M. oleifera* seed by coagulation, flocculation, sedimentation, and rapid granular filtration, in a pH range from 4.0 to 9.0 and a fixed coagulant dosage of 600 mg/L. It was observed that the zeta potential and turbidity removal efficiency decreased with increasing pH, and this was attributed to the isoelectric point of the cationic proteins being between 10 and 11, which reduced the treatment efficiency at values close to this pH.

Not all studied pH values were suitable for Aged-PVC MP and HA removal with alum. At pH 8.0, the tested alum dosages (up to 15 mg/L) failed to improve removal compared to the control (0 mg/L; Figure 6b,d,f), likely because of the predominance of negatively charged [Al­(OH)_4_]^−^ species that cannot neutralize negatively charged particles.^43^ Satisfactory results were obtained at pH 5.0–7.0, with lower dosages needed at lower pH because more positive hydrolyzed alum species were available.^43^ MOS-SE outperformed alum by delivering consistent removal of Aged-PVC MPs and NOM over a broader pH range (5.0–8.0 vs 5.0–7.0).

### 3.4 Ives Filterability Index under Varying pH and Coagulant Dosages

The Ives filterability index (IFI) for the MOS-SE and alum dosages (pH 5.0–8.0) after in-line filtration are shown in Figure 7. According to Ives (1979),^32^ optimal filterability is indicated by low-turbidity filtrates, and minimal increases in head loss at high filtration rates; thus, lower IFI values indicate greater efficiency of filtration. No absolute IFI cutoff exists, but under identical experimental conditions, a lower IFI indicates higher efficiency. High (poor) IFI values (0.2849–0.40407) were found without coagulant, indicating poor filtration. The performance improved markedly with increasing coagulant dosage for both MOS-SE and alum, with a consistently lower IFI for MOS-SE, particularly at pH 5.0 and 6.0. Per Ives (1979),^32^ the optimal coagulant dosage coincides with the lowest IFI: the lowest observed value was 0.00275 ± 0.00058 at 30 mg/L MOS-SE, pH 6.0, reflecting maximal efficiency. The alum IFI was less consistent, with no improvement over the control at a pH of 8.0. The trend of improving IFI with increasing coagulant dosage up to an optimum, followed by a deterioration in filterability at higher amounts, is consistent with previous research. Hunce et al. (2019),^44^ for instance, reported that an alum dosage of 16.7 mg/L yielded the minimum IFI for kaolin suspensions, after which the index began to increase again. This pattern suggests that overdosing the coagulant can lead to the formation of flocs that are less effectively retained, thereby compromising overall filter performance.

**Figure 7:**
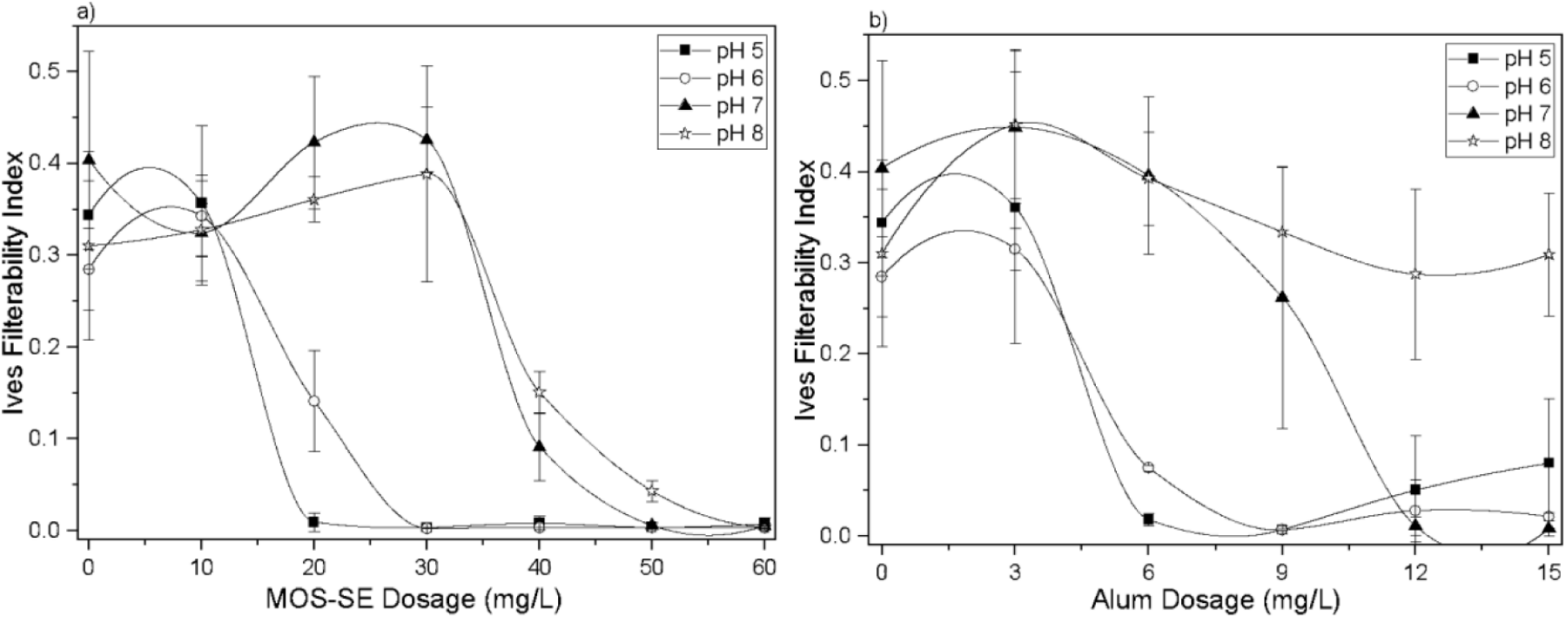
Ives filterability index after in-line filtration for Aged-PVC MPs and HA removal (pH 5.0–8.0) at various MOS-SE (a) and alum (b) dosages. The volumetric flow rate was 9.4 ± 2.2 cm/min. The error bars represent standard deviations.

The lowest alum IFI (0.0070 ± 0.00431) occurred at 9 mg/L and a pH of 6.0, which was statistically the same as the lowest value for the MOS-SE IFI. These optimal dosages coincide with maximum turbidity removals (Figure 1: 99.4 ± 0.1% for MOS-SE, 98.6 ± 0.8% for alum). Hunce et al. (2019)^44^ investigated the use of alum as a coagulant at varying dosages for removing turbidity from synthetic water prepared with kaolin via direct filtration. Their results revealed that in the absence of coagulant addition, the IFI values were high, indicating poor filtration performance. When alum was applied, the IFI markedly improved, with the best filtration efficiency occurring at a dosage of 16.7 mg/L alum, where turbidity removal was maximized and filterability was optimal under the experimental conditions. Similarly, Tchio et al. (2003)^45^ studied the effect of alum coagulant dosage on IFI during direct filtration of synthetic water containing suspended kaolin particles. Their results indicated that IFI values significantly decreased with increasing alum dosage, reflecting improved filterability. The lowest IFI, corresponding to the best filtration efficiency, was obtained at an alum dosage of 9 mg/L. These findings align with those of Hunce et al. (2019),^44^ who confirmed that alum dosage critically influences filtration performance metrics and that optimal dosing can significantly improve turbidity removal during direct filtration of kaolin-laden water.

### 3.5 Quantification of Aged-PVC MPs by Particle Counting under Optimized Conditions

SEM analyses were performed for synthetic water and coagulated-filtered samples under optimized in-line conditions (30 mg/L MOS-SE, 9 mg/L alum, pH 6.0) selected on the basis of prior residual turbidity results. The initial number of Aged-PVC MPs in synthetic water (15 mg/L Aged-PVC MPs and 10 mg/L HA in tap water) was 4.5 × 10^7^/L, which is consistent with prior SEM counts for PVC MPs at 10 mg/L (1.8 × 10^7^/L) reported previously.^30^

The particle count reductions of Aged-PVC MPs were 98.5% and 98.7% for MOS-SE and alum, respectively. Illustrative SEM images are shown in Figure 8. These removal efficiencies statistically match those obtained indirectly by turbidity reduction (Figure 1: 99.4 ± 0.1% for MOS-SE, 98.6 ± 0.8% for alum), confirming that turbidity-based indirect removal analysis is reliable.

**Figure 8:**
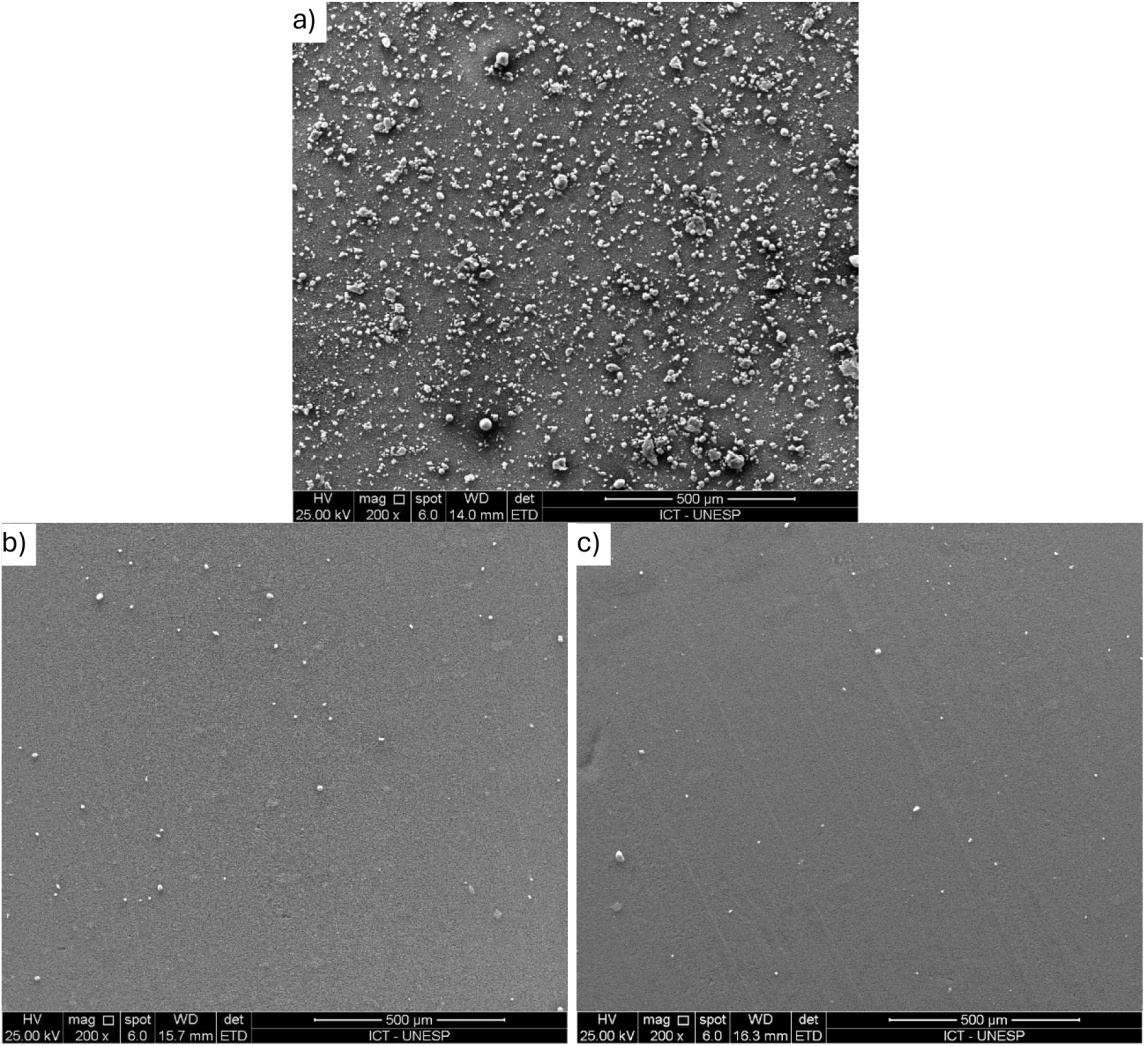
Representative SEM images: (a) synthetic water (15 mg/L Aged-PVC MPs and 10 mg/L HA in tap water), (b) after in-line filtration with MOS-SE (30 mg/L, pH 6.0), and (c) with alum (9 mg/L, pH 6.0).

SEM also revealed complete removal of Aged-PVC MPs >15 μm after in-line filtration, with the remaining MPs showing D50 values of 6.3 μm (MOS-SE) and 5.0 μm (alum). High-magnification SEM images of residual Aged-PVC MPs after filtration are shown in Figure 9. Consistent findings have been reported in the literature: Cherniak et al. (2022)^24^ reported the granular filtration removal of MPs at 94% (125–300 μm), 59% (45–125 μm), and 57% (10–45 μm), whereas Na et al. (2021)^37^ reported the complete removal of MPs >45 μm and 83.4% for 10 μm MPs, all of which are attributed to filter pore transport and adhesion mechanisms that are sensitive to MP particle size.

**Figure 9:**
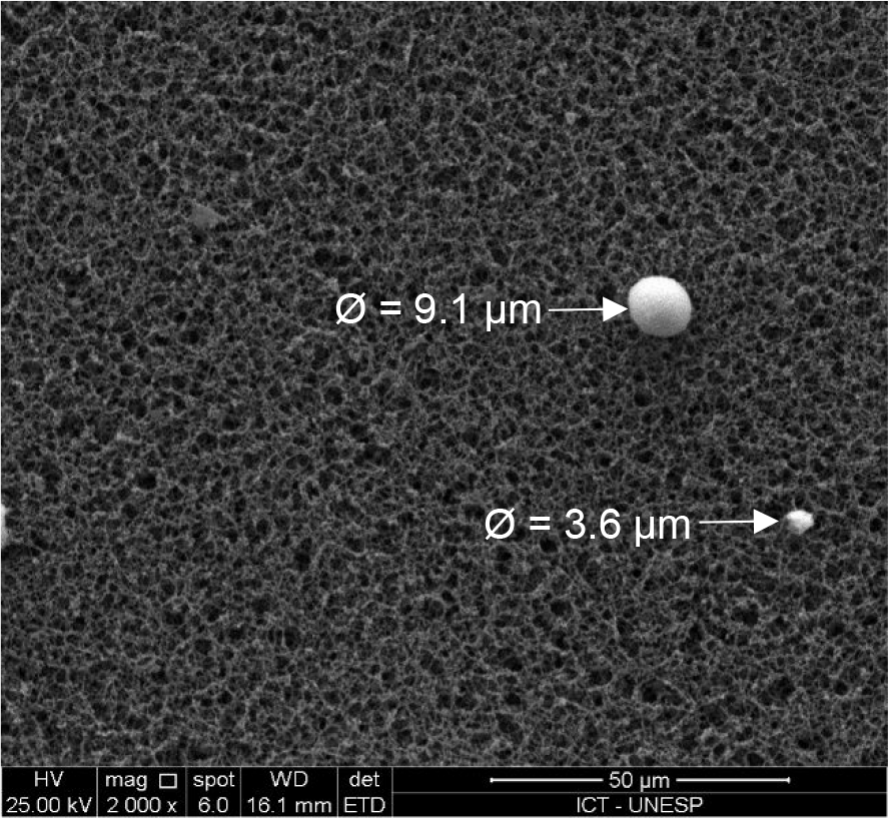
High-magnification SEM image (2000×) of residual Aged-PVC MPs after in-line filtration using MOS-SE (30 mg/L, pH 6.0).

### 3.6 Impact on SUVA after in-Line Filtration

The synthetic water SUVA, calculated from Table 1 and eq 1, was 8.5 ± 0.01 L/(mg·m), reflecting the high concentration of humic acid. SUVA values >4 indicate predominantly hydrophobic, aromatic, high-molecular-weight NOM; SUVA <3 denotes hydrophilic, nonhumic, low-molecular-weight fractions.^35^ High SUVA NOM increases chlorine demand and the formation of DBPs such as trihalomethanes (THMs), increasing health risks. Consequently, their removal during water treatment is crucial prior to the disinfection step.^46^

The DOC and SUVA values for treated water (in-line filtration, pH 6.0, varying MOS-SE dosage) are shown in Figure 10. Increasing the MOS-SE dosage increased the DOC. The use of crude *M. oleifera* seeds increases residual organic matter because of the seed composition, such as proteins, lipids and vitamins.^16,41,47^ Chales et al. (2022)^38^ evaluated different dosages of *M. oleifera* seed coagulant prepared by various extraction and defatting methods for the treatment of water contaminated with kaolin. Their results indicated that increasing the dosage of the coagulant correspondingly increased the dissolved organic carbon (DOC) concentration in the treated water, irrespective of the extraction method used. This effect is attributed to the organic matter introduced by the seed extracts themselves, which increases the DOC background posttreatment. Similarly, Andrade et al. (2021)^36^ reported a 104% increase in biochemical oxygen demand (BOD) during the tertiary treatment of domestic wastewater when 600 mg/L aqueous *M. oleifera* seed extract was applied as a coagulant. This increase was also attributed to residual organic matter originating from the seed extract.

**Figure 10:**
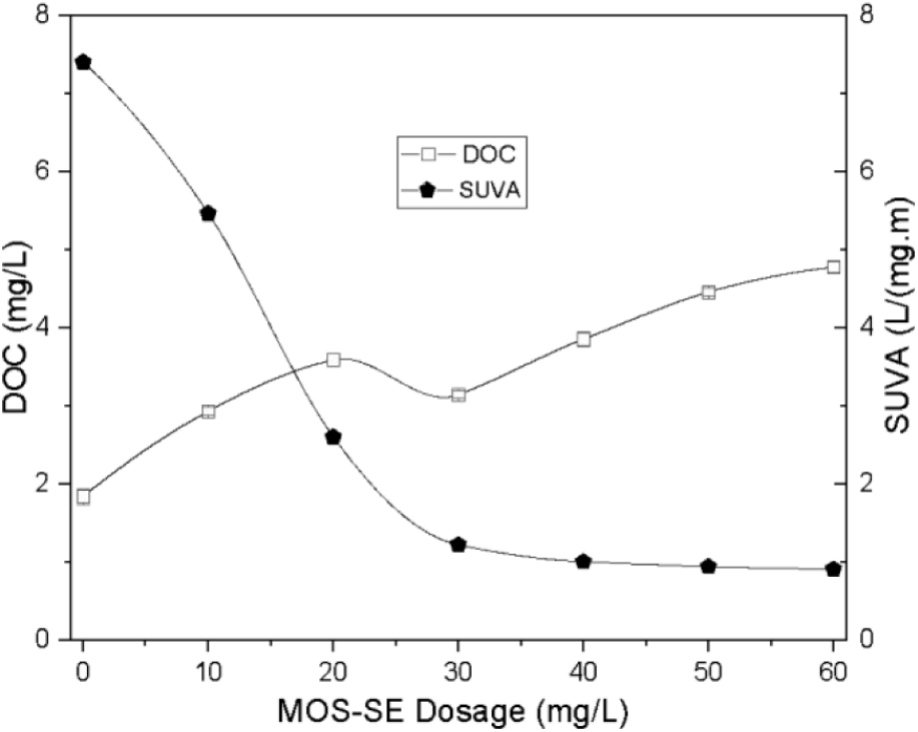
Dissolved organic carbon (DOC) and specific ultraviolet absorbance (SUVA) values for varying MOS-SE dosages at pH 6.0 after in-line filtration. The error bars represent standard deviations.

Conversely, increasing the MOS-SE dosage substantially decreased the SUVA (Figure 10), which decreased below 1 L/(mg·m) for dosages >30 mg/L, an 88% decrease in the SUVA compared with that of synthetic water, demonstrating the removal of the hydrophobic NOM fractions (humic acid) by MOS-SE coagulation and rapid granular filtration. Hydrophobic NOM, with a high negative charge, is more susceptible to coagulation than hydrophilic fractions.^35^ Low SUVA values obtained after MOS-SE treatment thus confirm the removal of most humic substances, whereas hydrophilic low-molecular-weight NOM (proteins, polysaccharides, and amino acids) is recalcitrant.^48^ This residual hydrophilic fraction of NOM is attributable to the seed itself. Baptista et al. (2017)^49^ reported a 4.6-fold increase in DOC and an 83% decrease in SUVA with *M. oleifera* seed extracts used to treat water from rivers. Okoro et al. (2021)^50^ reported up to 92% SUVA reduction with kenaf seed extract but also up to 2.5-fold increased DOC when natural superficial water was treated, both of which are attributable to the organic nature of natural coagulants and their efficiency for removing hydrophobic NOM.

## 4 Conclusion

This investigation demonstrated that *Moringa oleifera* seed saline extract (MOS-SE) represents a viable and sustainable alternative to conventional aluminum-based coagulants for removing PVC MPs from drinking water. The key findings establish that MOS-SE achieves removal efficiency (>98%) comparable to that of alum while operating effectively across a broader pH range, offering significant operational advantages for water treatment facilities. The demonstration that in-line filtration performs equivalently to direct filtration eliminates the need for energy-intensive flocculation, reducing both capital and operational costs.

Critically, this work addresses a significant knowledge gap in PVC MPs remediation by quantitatively evaluating natural coagulants in rapid granular filtration systems, configurations widely employed in drinking water treatment but previously understudied for this emerging contaminant. The integration of nonintrusive floc imaging with conventional performance metrics provides mechanistic insights into particle destabilization and retention processes.

MOS-SE demonstrated substantial efficacy in decreasing SUVA values (88%), indicating efficient removal of aromatic NOM components from treated water, thereby minimizing the formation potential of trihalomethanes during subsequent chlorination processes. However, this study reveals important limitations of natural coagulants, particularly the increase in dissolved organic carbon, which may complicate downstream treatment processes. Future research should focus on seed purification methods to minimize the leaching of organic matter while maintaining coagulant efficacy. Furthermore, while this study confirms the utility of the IFI as a performance metric, additional research could further validate its application for optimizing coagulant selection for different types of MPs and complex water matrices, such as natural water, strengthening its role as a practical tool for treatment plant operators.

These findings contribute essential data to the emerging field of sustainable water treatment technologies and provide a foundation for future studies of the role of natural coagulants in PVC MP removal.

## Supplementary Material



## Notes

Particle size distribution curve of Aged-PVC MPs; correlation curve between turbidity and Aged-PVC MP concentration; image of the jar test apparatus used in the experiments; image of the setup used for nonintrusive floc imaging; graph showing the effect of 1 M NaCl on the removal of the studied parameters (PDF)
