# Supplementary material for: Removal of Microplastics from Drinking Water by Moringa oleifera Seed: Comparative Performance with Alum in Direct and in-Line Filtration Systems
Source: ACS Omega. 2026 Jan 19;11(4):6602–12. doi: 10.1021/acsomega.5c11569 (PMC12878436; doi:10.1021/acsomega.5c11569)
Supplement: Supplementary file 1 [file ao5c11569_si_001.pdf]

Supporting Information

Removal of Microplastics from Drinking Water by *Moringa oleifera* Seed: Comparative Performance with Alum in Direct and In-Line Filtration Systems

Gabrielle S. Batista, Victoria A. S. Ferreira, Luiz G. R. Godoy, Rodrigo B. Moruzzi, Soroosh Sharifi, and Adriano G. dos Reis*

AUTHOR INFORMATION

*Corresponding author:

Adriano G. dos Reis - São Paulo State University (UNESP), Institute of Science and Technology, Environmental Engineering Department, São José dos Campos, 12247-016, Brazil, https://orcid.org/0000-0001-6465-4538; phone: +55-12-3947-9721; E-mail: adriano.reis@unesp.br

Authors:

Gabrielle S. Batista - São Paulo State University (UNESP), Institute of Science and Technology, Environmental Engineering Department, São José dos Campos, 12247-016, Brazil, https://orcid.org/0009-0008-3575-8811

Victoria A. S. Ferreira - São Paulo State University (UNESP), Institute of Science and Technology, Environmental Engineering Department, São José dos Campos, 12247-016, Brazil, https://orcid.org/0000-0003-4503-7416

Luiz G. R. Godoy - São Paulo State University (UNESP), Institute of Science and Technology, Environmental Engineering Department, São José dos Campos, 12247-016, Brazil, https://orcid.org/0000-0002-1466-9042

Rodrigo B. Moruzzi - São Paulo State University (UNESP), Institute of Science and Technology, Environmental Engineering Department, São José dos Campos, 12247-016, Brazil, https://orcid.org/0000-0002-1573-3747

Soroosh Sharifi - University of Birmingham, School of Engineering, Department of Civil Engineering, Edgbaston, Birmingham, B15 2TT, United Kingdom, https://orcid.org/0000-0003-0717-1729

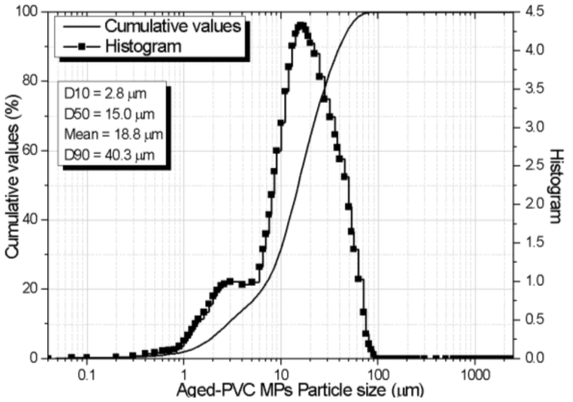

Fig. S1 – Particle size distribution of Aged-PVC MP powder particles

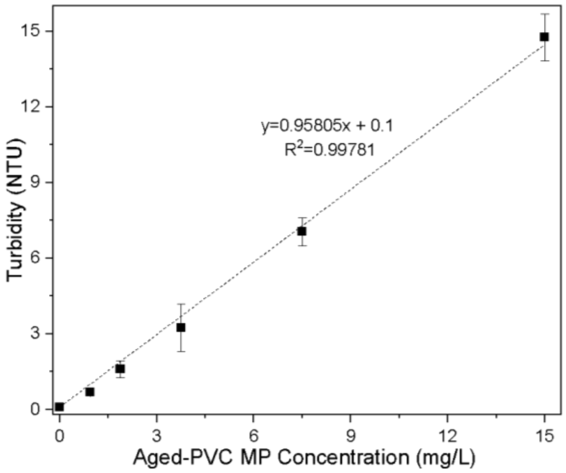

Fig. S2 – Concentration–turbidity relationships of Aged-PVC MP and HA suspensions in tap water. The error bars represent standard deviation values.

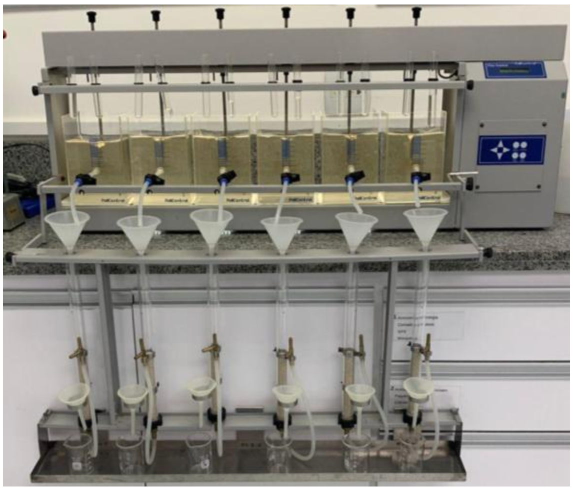

Fig. S3 – Bench-scale coagulation–flocculation jar test coupled with a rapid sand filtration column to simulate the sequential unit operations of coagulation, flocculation, and rapid granular filtration.

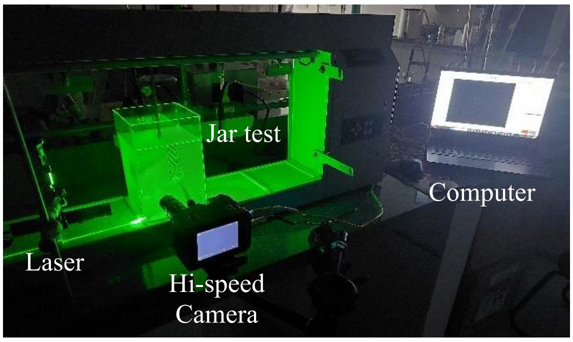

Fig. S4 – Photograph of the experimental setup used for nonintrusive image acquisition to measure the floc aggregate size.

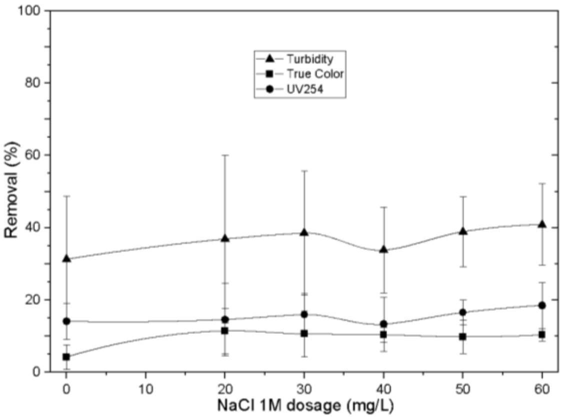

Fig. S5 – Removal efficiencies of turbidity, true color, and UV_{254} when 1 M NaCl was used as a coagulant at various dosages after inline filtration at pH 6.0. The error bars represent the standard deviations
